# Short-term toxicity studies of thallium (I) sulfate administered in drinking water to Sprague Dawley rats and B6C3F1/N mice

**DOI:** 10.1016/j.toxrep.2023.05.003

**Published:** 2023-05-11

**Authors:** Kelly A. Shipkowski, Troy D. Hubbard, Kristen Ryan, Suramya Waidyanatha, Helen Cunny, Keith R. Shockley, Joshua L. Allen, Heather Toy, Keith Levine, James Harrington, Laura Betz, Barney Sparrow, Georgia K. Roberts

**Affiliations:** a Division of Translational Toxicology, National Institute of Environmental Health Sciences, Research Triangle Park, NC, USA; b Division of Intramural Research, National Institute of Environmental Health Sciences, Research Triangle Park, NC, USA; c Battelle Memorial Institute, Columbus, OH, USA; d RTI International, Discovery Sciences, Research Triangle Park, NC, USA; e Social & Scientific Systems, a DLH Company, Research Triangle Park, NC, USA

**Keywords:** Thallium (I) sulfate, Thallium salts, Dose range-finding study, Internal concentration assessment, Alopecia

## Abstract

Thallium is a heavy metal that is known to induce a broad spectrum of adverse health effects in humans including alopecia, neurotoxicity, and mortality following high dose acute poisoning events. Widespread human exposure to thallium may occur via consumption of contaminated drinking water; limited toxicity data are available to evaluate the corresponding public health risk. To address this data gap, the Division of Translational Toxicology conducted short-term toxicity studies of a monovalent thallium salt, thallium (I) sulfate. Thallium (I) sulfate was administered via dosed drinking water to time-mated Sprague Dawley (Hsd:Sprague Dawley® SD®) rats (F_0_ dams) and their offspring (F_1_) from gestation day (GD) 6 until up to postnatal day (PND) 28 at concentrations of 0, 3.13, 6.25, 12.5, 25, or 50 mg/L, and adult male and female B6C3F1/N mice for up to 2 weeks at concentrations of 0, 6.25, 12.5, 25, 50, or 100 mg/L. Rat dams in the 50 mg/L exposure group were removed during gestation, and dams and offspring in the 25 mg/L exposure group were removed on or before PND 0 due to overt toxicity. Exposure to thallium (I) sulfate at concentrations ≤ 12.5 mg/L did not impact F_0_ dam body weights, maintenance of pregnancy, littering parameters, or F_1_ survival (PND 4–28). However, in F_1_ pups, exposure to 12.5 mg/L thallium (I) sulfate resulted in decreased body weight gains relative to control rats and onset of whole-body alopecia. Measurement of thallium concentrations in dam plasma, amniotic fluid, fetuses (GD 18), and pup plasma (PND 4) indicated marked maternal transfer of thallium to offspring during gestation and lactation. Mice exposed to 100 mg/L thallium (I) sulfate were removed early due to overt toxicity, and mice exposed to ≥ 25 mg/L exhibited exposure concentration-related decreases in body weight. Lowest-observed-effect levels of 12.5 mg/L (rats) and 25 mg/L (mice) were determined based on the increased incidence of clinical signs of alopecia in F_1_ rat pups and significantly decreased body weights for both rats and mice.

## Highlights

• Exposure to thallium (I) sulfate results in decreased body weight in rats and mice.
• Thallium (I) sulfate exposure induces whole-body alopecia in rats, but not mice.
• Identified LOELs were 12.5 mg/L (rats) and 25 mg/L (mice).
• These data will aid in evaluating the potential health risk of thallium (I) sulfate.

## 1 Introduction

Thallium is a heavy metal that is soft, malleable, and insoluble in water in its metallic state. In soil, the natural abundance of thallium can vary on the order of 0.1–1 ppm; higher concentrations can occur in the vicinity of metallic ore deposits [1]. Thallium and thallium salts (monovalent [Tl^+1^] or trivalent [Tl^+3^]) have been used in a variety of industries, including the production of semiconductors, electronics, fireworks, and imitation gems. Anthropogenic sources of thallium pollution include gaseous emissions from cement factories and coal burning power plants [1]. Additionally, leaching of thallium into drinking water sources can occur as a result of ore processing methods used in the mining industry. The U.S. Environmental Protection Agency (EPA) Toxics Release Inventory estimated that approximately 10,200 pounds of thallium and approximately 1,000,000 pounds of thallium compounds were released into the environment from industrial sources in 2021 [2].

There is potential for widespread human exposure to thallium due to its presence as a contaminant in drinking water in the vicinity of hazardous waste and industrial sites or consumption of food crops grown in contaminated soil [3], [4], [5]. Information from the Environmental Working Group’s 2017–2019 tap water database showed that thallium had been detected in treated tap water in 40 states, exposing an estimated 9.8 million people, with four of the tested utilities exceeding the current U.S. EPA maximum contaminant level for thallium in drinking water of 2.0 ppb [6]. Urinary thallium levels in the U.S. population have been measured as a part of the National Health and Nutrition Examination Survey conducted by the National Center for Health Statistics. Measurable urinary levels of thallium were detected in most survey participants; the 2015–2016 survey cycle reported creatinine-adjusted geometric mean and 95th percentile concentrations for all participants as 0.171 μg/g creatinine and 0.432 μg/g creatinine, respectively [7]. Notably, higher urinary thallium levels were detected in participants ages 3–5 (creatinine-adjusted geometric mean: 0.344 μg/g creatinine, 95th percentile: 0.850 μg/g creatinine), suggesting higher exposure in children [7].

Thallium toxicity is well characterized in humans due to reported cases of accidental or deliberate poisoning events. Tl^+1^ compounds are readily absorbed in the gastrointestinal tract. Historical use of thallium as a depilatory agent and topical treatment for ringworm suggests that it is absorbed dermally [8], [9], [10]. In humans, the urinary excretion half-life of thallium is estimated at 21.7 days [11]. Thallium has the potential to induce a broad spectrum of adverse health effects in humans, including alopecia (hair loss), neurological and cardiovascular effects, and mortality at high doses [12], [13], [14], [15], [16], [17], [18], [19], [20], [21]. In adults, lethal oral doses have been estimated at 10–15 mg thallium/kg body weight [22], [10]. There are indications in the literature that thallium is a neurotoxicant that targets the peripheral and central nervous systems following oral exposure with case studies in humans noting significant effects, including paresthesia, numbness, loss of vision, slurred speech, and polyneuropathy [13], [14], [15], [17], [20]. Of these case reports, the lowest single dose that induced negative health effects was reported by [14] at 0.31 g thallium (I) acetate, or approximately 3.4 mg/kg thallium [14], [10]. Following cessation of exposure symptoms may improve; however, indications of peripheral and central nervous system toxicity may be permanent [12], [18], [9], [20]. In many cases, diagnosis of thallotoxicosis is not established prior to the onset of the hallmark symptom of alopecia, which may present one to two weeks following exposure or onset of neurological abnormalities, delaying initiation of therapeutic intervention. Analyses of multiple case reports of women exposed to thallium during pregnancy suggest a possible association with premature, low-birth-weight infants [23].

Animal studies support many of the toxicological findings reported in humans. Alopecia, neurological abnormalities, kidney effects, and mortality have been observed in rats following oral administration of thallium compounds. Female Sprague Dawley rats exposed to thallium in drinking water containing 10 mg/L thallium (I) sulfate, equivalent to 1.4 mg thallium/kg/day, presented with alopecia following approximately four weeks of exposure [24]. In the same study, histopathological alterations in peripheral nerves, including changes in the sciatic myelin sheath, axonal degeneration, mitochondrial degeneration, neurofilamentous clustering, and increased lysosomal activity, were noted [24]. Some studies suggest that thallium exposure may impact male reproductive function, and rodents orally exposed to thallium compounds have presented with histological alterations of the testes and effects on sperm parameters [25], [26], [27]. While the exact mechanism of thallium-induced toxicity is unknown, it has been established that Tl^+1^ can replace potassium (K^+^) in vital biological pathways. Tl^+1^ is similar to K^+^ in ionic radius and valence charge, and Tl^+1^ has a ten-fold higher affinity for rabbit kidney Na^+^/K^+^ ATPase relative to K^+^ [28]. The putative mechanism of thallium toxicity is thought to involve inhibition of the Na^+^/K^+^ ATPase pump, depolarization of membranes, disturbance of mitochondrial function, and/or oxidative stress.

A 2009 EPA Integrated Risk Information System assessment concluded that there was inadequate subchronic or chronic mammalian toxicity data to derive an oral reference dose or inhalation reference concentration for lifetime thallium exposure that would be without appreciable risk to human health [10]. In many cases, past studies have utilized acute exposure methods to mimic instances of poisoning; however, prolonged low-level exposure better corresponds to exposure via contaminated drinking water occurring in human populations. In response to the paucity of toxicological data available for thallium compounds, thallium (I) sulfate was selected for evaluation as a representative monovalent thallium compound. The short-term toxicity studies described herein are part of an expanded research program (https://ntp.niehs.nih.gov/go/ts-16019) aimed at characterizing the hazard associated with exposure to thallium compounds. Current studies described here utilized a drinking water exposure paradigm to mimic the likely route of human thallium intake and were conducted to (1) provide preliminary information on potential toxicity targets, and (2) inform exposure concentration selection for follow-up subchronic studies of thallium toxicity.

## 2 Materials and methods

### 2.1 Chemical characterization and dose formulation

Thallium (I) sulfate (CASRN 7446–18–6; lot #18783100), was purchased from Strem Chemicals, Inc. (Newburyport, MA). The purity and identity of the test article were determined by ion chromatography (IC; sulfate content) and inductively coupled plasma-optical emission spectrometry (ICP-OES; thallium content). The identity of the test article was confirmed as thallium (I) sulfate and contained approximately 18.6% sulfate and 80.3% thallium, which were approximately 98.0% and 99.1% of the theoretical values for sulfate and thallium in thallium (I) sulfate, respectively.

Drinking water formulations (0, 3.13, 6.25, 12.5, 25, 50, and 100 mg/L) of thallium (I) sulfate (∼pH 5.7) were prepared using deionized (DI) water. Formulations were analyzed utilizing validated analytical methods using IC (sulfate content) and ICP-OES (thallium content) [linearity (r)> 0.99; relative error ≤ ± 3.4%; relative standard deviation ≤ 0.8%] prior to study start and following the last exposure; the thallium (I) sulfate content of all samples was within 10% of the target concentration. The sulfate and thallium content of the vehicle control formulation (0 mg/L) were below the limit of quantitation, with respect to formulation concentration (sulfate = 0.10 mg/L, thallium = 0.3 mg/L). Prior to study start, stability of the thallium (I) sulfate formulations (3.95 mg/L) in DI water was confirmed for up to 42 days when stored in clear plastic containers at room temperature (∼25 °C) or refrigerated (∼5 °C). Additionally, analyses of test article speciation found no formation of Tl^+3^ under the same storage conditions.

### 2.2 Animals and animal maintenance

These studies were conducted at Battelle (West Jefferson, OH). Time-mated (F_0_) Sprague Dawley (Hsd:Sprague Dawley®SD®) rats were obtained from Envigo (Haslett, MI), and adult (3–4 weeks old) B6C3F1/N mice were obtained from the National Toxicology Program (NTP) colony maintained by Taconic Biosciences, Inc. (Germantown, NY). Upon arrival, animals were quarantined for 14 days (rats) or 12 days (mice). Rats and mice were housed in polycarbonate cages containing irradiated Sani-Chips® hardwood bedding (PJ Murphy Forest Products Corp., Montville, NJ), with ad libitum access to irradiated NIH-07 wafer feed (rats; Zeigler Brothers, Gardners, PA) or irradiated NTP-2000 pellet feed (mice; Zeigler Brothers, Gardners, PA). Rat dams were housed individually except during lactation when they were housed with their respective litter. Male mice were housed individually, and females were housed up to five per cage. Prior to thallium (I) sulfate exposure, all animals had ad libitum access to tap water from the Village of West Jefferson (West Jefferson, OH) water supply.

Animal use was in accordance with the U.S. Public Health Service Policy on Humane Care and Use of Animals. All animal studies were conducted in an animal facility accredited by AAALAC International. Studies were approved by the Battelle (Columbus, OH) Animal Care and Use Committee and conducted in accordance with all relevant National Institutes of Health and NTP animal care and use policies and applicable federal, state, and local regulations and guidelines. These studies were conducted in compliance with the U.S. Food and Drug Administration Good Laboratory Practice Regulations [29].

### 2.3 Perinatal study in rats

Time-mated F_0_ female rats (n = 12/group) were provided ad libitum access to dosed drinking water containing thallium (I) sulfate at concentrations of 0, 3.13, 6.25, 12.5, 25, or 50 mg/L. An additional 8 dams were included in the 0, 6.25, and 25 mg/L groups for internal concentration assessment. Exposure was initiated on gestation day (GD) 6 and continued until termination or postnatal day (PND) 28. The GD 6 – PND 28 exposure interval covers all developmental stages from implantation through birth and subsequent weaning, allowing for a complete assessment of effects following in utero exposure. GD 0 was designated as the day in which mating status was confirmed by the presence of a copulatory plug. Animals were observed for mortality and morbidity twice daily and out of cage clinical observations were performed once daily. Dam body weights were recorded upon arrival and on GDs 3, 5, 6, 9, 12, 15, 18, and 21. Dam and pup body weights were recorded on PNDs 1, 4, 7, 10, 13, 16, 19, 21, 25, and 28. Litter weights, by sex, and total litter weight were also recorded on PND 1. Dam water consumption measurements were initiated on GD 6 and recorded on GDs 9, 12, 15, 18, and 21. Water consumption measurements by cage (dam + offspring) were initiated on PND 1 and recorded on PNDs 4, 7, 10, 13, 16, 19, 21, 23, 25, 27, and 28. Following parturition, the number of live and dead pups of each sex was recorded on PNDs 1, 4, 7, 10, 13, 16, 19, 21, 25, and 28. The sex of live and dead pups was determined beginning at PND 1 by visually assessing the distance between the genital tubercle and the anus (anogenital distance), which is greater in males than in females, and confirmatory physical characteristics such as the presence of testes or vaginal opening at later ages.

### 2.4 2-week study in adult mice

Male and female B6C3F1/N mice (n = 5/sex/group) were provided ad libitum access to dosed drinking water containing thallium (I) sulfate at concentrations of 0, 6.25, 12.5, 25, 50, or 100 mg/L for two weeks. Animals were observed for mortality and morbidity twice daily and clinical observations of toxicity once daily. Body weights were recorded prior to study start and twice weekly thereafter. Water consumption measurements began on study day (SD) 0 and were recorded twice weekly. At study termination (SD 14), surviving male and female mice were subjected to complete necropsies and gross examination of tissues. Organ weights were recorded for the liver, thymus, kidney, testis, epididymis, ovary, heart, and lungs; all bilateral organs were weighed separately.

### 2.5 Internal concentration assessment

#### 2.5.1 Sample collection

Rat dams and their fetuses/pups in select groups were removed on GD 18, PND 4, and PND 28 for assessment of internal thallium concentrations. The allocation of dams and litters for internal concentration assessment is displayed in Table 1. On GD 18, plasma, red blood cells (RBCs), amniotic fluid, and fetuses were collected from 3 dams/group in the 0, 6.25, and 25 mg/L groups. On PND 4, plasma and RBC samples were collected from dams and their pups (n = 3/group) in the 0 and 6.25 mg/L groups. On PND 28, plasma and RBCs were collected from dams and their pups (n = 5/group) in the 0, 3.13, 6.25, and 12.5 mg/L groups.

**Table 1:** Survival and Disposition of Dams in the Perinatal Rat Study of Thallium (I) Sulfate.

	0 mg/L	3.13 mg/L	6.25 mg/L	12.5 mg/L	25 mg/L	50 mg/L
Animals Initially on Study	20	12	20	12	20	12
Biological Sampling^a^						
GD 18	3	---	3	---	3	---
PND 4	3	---	3	---	---b	---
PND 28	5	5	5	5	---	---
Euthanized Moribund	0	0	0	0	5	3
Found Dead	0	0	0	0	3	2
Removed from Study	0	0	0	0	9	7
Surviving to Study Termination^c^	9 (100%)	7 (100%)	9 (100%)	7 (100%)	0 (0%)	0 (0%)

GD = gestation day; PND = postnatal day.

a Includes animals utilized for internal thallium concentration assessments.

b Due to observations of moribundity this group was removed from study prior to PND 4.

c The number of animals surviving to study termination excludes animals utilized for biological sampling on PND 28. Percentages calculated as [animals surviving to study termination/(animals initially on study – animals used for biological sampling)].

Dams and pups were euthanized by decapitation; fetuses were also euthanized via decapitation upon removal by caesarian section. Blood was collected into K_3_EDTA tubes from dams and pups via trunk blood collection immediately following euthanasia.

All blood samples were collected in the morning as soon as possible after the lights came on, and all samples on a particular day were collected within a 2-hour window; each sample was frozen within 2 hours of collection. Blood samples were maintained on wet ice at collection and centrifuged within one hour of collection for plasma and RBCs. RBCs were washed 3 times with normal saline (approximately twice the volume of the RBCs) and isolated as one sample per animal. Amniotic fluid and fetuses were pooled by litter. All samples were flash frozen in liquid nitrogen and stored frozen at − 85 to − 60 °C.

On SD 14, plasma and RBCs were collected from mice (n = 5/sex/group) in the 0, 6.25, 12.5, 25, and 50 mg/L groups. Blood was collected from the cranial vena cava of anesthetized (70% CO_2_: 30% O_2_) mice into K_3_EDTA tubes. All blood samples were collected and processed following the same procedures described for rats.

#### 2.5.2 Sample analysis

Thallium concentrations in biological samples were quantified using a qualified inductively coupled plasma-mass spectrometry (ICP-MS) method (see Supplemental Material).

### 2.6 Statistical methods

Statistical methods were chosen based on distributional assumptions as well as the need to incorporate within-litter correlation among rats in the perinatal toxicity study. Unless specifically mentioned, all endpoints were tested for a trend across exposure groups, followed by pairwise tests for each exposure group against the control group. Significance of all trend and pairwise tests is reported at both 0.05 and 0.01 levels. Statistical analyses were performed using SAS 9.4 (SAS Institute, Cary, NC). For continuous endpoints, extreme values were identified by the outlier test of Dixon and Massey [30] for small samples (n < 20) regardless of presence of littermates. For endpoints with large samples (n ≥ 20) and no littermates, Tukey’s outer fences method [31] was used. For endpoints with large samples and where littermates were present, all observations across exposure groups were fit to a linear mixed effects model with a random litter effect, and the residuals were tested by exposure group for outliers using Tukey’s outer fences method. All flagged outliers were examined, and implausible values were eliminated from the final analysis.

For the perinatal study in rats, pup body weights were adjusted for live litter size on PND 1 and analyzed for exposure concentration effects using a linear mixed model with litter as random effect. To adjust for multiple comparisons, a Dunnett-Hsu adjustment was used [32]. Dam body weights during gestation and lactation were analyzed with the parametric multiple comparison procedures of Dunnett or Williams, depending on whether Jonckheere’s test indicated the use of a trend-sensitive test [33], [34], [35], [36]. Litter size, survival, water consumption, and gestational length, which typically have skewed distributions, were analyzed using the nonparametric multiple comparison methods of Dunn or Shirley (as modified by Williams and Dunn) [37], [38], [39]. Jonckheere’s test was used to assess the significance of exposure concentration-related trends and to determine whether a trend-sensitive test (the Williams or Shirley test) was more appropriate for pairwise comparisons than a test that does not assume a monotonic exposure concentration-related trend (Dunnett’s or Dunn’s test). Cochran-Armitage trend tests were used to test the significance of trends in gestational and fertility data across exposure groups [40]. Fisher’s exact test was used to conduct pairwise comparisons of each exposure group with the control group. P values for all analyses were two-sided.

For the 2-week study in B6C3F1/N mice, organ and body weight data, which historically have approximately normal distributions, were analyzed with the Dunnett or Williams test. Water consumption data were analyzed using the nonparametric multiple comparison methods of Dunn or Shirley (as modified by Williams and Dunn). Jonckheere’s test was used to assess the significance of exposure concentration-related trends and to determine whether a trend-sensitive test (the Williams or Shirley test) was more appropriate for pairwise comparisons than a test that does not assume a monotonic exposure concentration-related trend (Dunnett’s or Dunn’s test).

With the exception of PND 4 pups, tissue concentration data for rats and mice were analyzed by Jonckheere’s (trend) and Shirley’s or Dunn’s (pairwise) tests. Pairwise comparisons of pup PND 4 concentration data were performed using the Datta-Satten modified Wilcoxon test that incorporated litter effects [41]. For situations where only two exposure groups were present, no trend test was performed, and no multiple comparisons adjustment was applied. For non-litter effects cases with only two exposure groups the Shirley/Dunn test is equivalent to a Wilcoxon test.

## 3 Results

All summary and individual animal data from the toxicological evaluations described here are publicly available via the National Toxicology Program’s Chemical Effects in Biological Systems (CEBS) database: https://doi.org/10.22427/NTP-DATA-500-003-001-000-9. All data not reported here can be found as supplemental data in CEBS.

### 3.1 Perinatal toxicity study in rats

#### 3.1.1 Maternal viability and clinical observations

Mortality attributed to thallium (I) sulfate exposure occurred in F_0_ dams in the 25 and 50 mg/L groups (Table 1). Three dams in the 50 mg/L group were euthanized moribund (GD 14–16) and an additional two dams were found dead (GD 15–16). Five dams in the 25 mg/L group were euthanized moribund (GD 20–21) with three additional dams found dead (GD 20–22). Due to the observed overt toxicity, all remaining F_0_ dams and litters in the 25 mg/L group were humanely euthanized on GD 22 and PND 0, respectively, and dams in the 50 mg/L group were humanely euthanized on GD 16 (Table 1). All remaining dams in the 0, 3.13, 6.25, and 12.5 mg/L groups survived to study termination at PND 28 (Table 1).

Clinical observations during gestation were notable in the 25 and 50 mg/L dams (Table 2). Exposure-related observations were noted beginning on GD 13 (50 mg/L) and GD 17 (25 mg/L), and included labored breathing, cold to touch, eye/nasal discharge, soft feces, wet urogenital area, lethargy, paleness, and piloerection. These observations generally coincided with indications of overt toxicity or were observed prior to mortality in these groups. Regional alopecia of the dorsum, forelimb, hindlimb, or head was observed beginning on GD 6 in the 25 mg/L dams. Additional clinical observations of alopecia of the dorsum in the 12.5 mg/L dams and soft feces in all remaining groups were observed during lactation (Table 2).

**Table 2:** Clinical Observations in Rat Dams Exposed to Thallium (I) Sulfate during Gestation and Lactation^a^.

	0 mg/L	3.13 mg/L	6.25 mg/L	12.5 mg/L	25 mg/L	50 mg/L
**Gestation**						
Alopecia, Dorsum	0	0	0	0	1/20 (GD 18)	0
Alopecia, Forelimb	0	0	0	0	5/20 (GD 19)	0
Alopecia, Head	0	0	0	0	2/20 (GD 20)	0
Alopecia, Hindlimb	0	0	0	0	1/20 (GD 6)	0
Breathing, Labored	0	0	0	0	2/20 (GD 22)	2/12 (GD 14)
Cold to Touch	0	0	0	0	2/20 (GD 21)	3/12 (GD 14)
Discharge, Eye or Nose	0	0	3/20 (GD 6)	0	13/20 (GD 19)	9/12 (GD 14)
Feces, Soft	0	0	0	0	19/20 (GD 17)	12/12 (GD 13)
Lethargic	0	0	0	0	4/20 (GD 21)	3/12 (GD 15)
Pale	0	0	0	0	5/20 (GD 21)	10/12 (GD 14)
Piloerection	0	0	0	0	3/20 (GD 21)	3/12 (GD 14)
Urogenital Area, Wet	0	0	0	0	15/20 (GD 17)	12/12 (GD 13)
**Lactation**						
Alopecia, Dorsum	0	0	0	2/12 (LD 6)	1/4 (LD 0)	---^b^
Alopecia, Forelimb	0	0	0	0	2/4 (LD 0)	---
Alopecia, Head	0	0	0	0	1/4 (LD 0)	---
Feces, Soft	3/17 (LD 12)	6/12 (LD 4)	2/17 (LD 11)	6/12 (LD 9)	3/4 (LD 0)	---

GD = gestation day; LD = lactation day.

^a^Data are presented as the number of animals observed with clinical finding/the number of animals examined (the first day the clinical finding was observed).

^b^Data not available due to early removal of this group.

#### 3.1.2 Maternal water consumption and chemical intake

Gestational water consumption (g/animal/day) by F_0_ dams in all exposure groups not removed from the study was generally similar to that of control rats; however, there was a small but significant negative trend in water consumption over the GD 6–21 period (Table 3). Chemical intake [mg thallium (I) sulfate/kg/day], estimated from water consumption, was proportional to exposure concentration over the GD 6–21 period despite the small negative trend in water consumption (Table 3). The estimated daily dose over the GD 6–21 interval was 0, 0.4, 0.8, 1.6, and 3.2 mg thallium (I) sulfate/kg/day for the 0, 3.13, 6.25, 12.5, and 25 mg/L groups, respectively.

**Table 3:** Water Consumption and Thallium (I) Sulfate Intake from Drinking Water by Rat Dams during Gestation and Lactation.

	0 mg/L	3.13 mg/L	6.25 mg/L	12.5 mg/L	25 mg/L
Water Consumption (g/animal/day)^a^					
Gestation Interval					
GD 6–9	32.7 ± 1.2 (20)	33.1 ± 1.1 (12)	32.8 ± 0.8 (20)	31.9 ± 0.9 (12)	32.0 ± 0.9 (20)
GD 9–12	34.9 ± 1.0 * (20)	34.9 ± 1.0 (12)	35.1 ± 0.9 (20)	33.8 ± 1.2 (12)	33.3 ± 1.0 (20)
GD 12–15	37.7 ± 1.2 ** (20)	37.4 ± 0.7 (12)	37.0 ± 1.1 (20)	35.3 ± 1.1 (12)	34.7 ± 1.0 (20)
GD 15–18	46.8 ± 2.0 (20)	46.6 ± 1.0 (12)	47.6 ± 1.3 (20)	44.8 ± 2.0 (12)	49.2 ± 1.7 (20)
GD 18–21	45.5 ± 1.9 * (17)	45.6 ± 1.4 (12)	44.3 ± 1.1 (17)	44.6 ± 1.9 (12)	38.1 ± 2.4 (15)
GD 6–21	40.0 ± 1.5 * (17)	39.5 ± 0.8 (12)	39.2 ± 1.0 (17)	38.1 ± 1.3 (12)	36.8 ± 1.0 (15)
**Lactation Interval**^**b**^					
LD 1–4	52.0 ± 1.3 * (17)	51.4 ± 1.2 (12)	52.9 ± 1.5 (17)	57.6 ± 1.9 (12)	---^c^
LD 4–7	66.2 ± 2.3 * (14)	71.0 ± 2.2 (12)	70.1 ± 2.2 (14)	75.0 ± 2.3 * (12)	---
LD 7–10	81.8 ± 2.7 * (14)	85.7 ± 2.1 (12)	84.3 ± 2.7 (14)	91.0 ± 2.0 * (12)	---
LD 10–13	93.6 ± 3.4 * (14)	98.2 ± 2.8 (12)	95.6 ± 2.4 (14)	104.9 ± 1.8 * (12)	---
LD 1–13	73.2 ± 2.1 ** (14)	76.6 ± 1.7 (12)	75.8 ± 2.0 (14)	82.1 ± 1.7 ** (12)	---
**Thallium (I) Sulfate Intake (mg/kg/day)**^**d**^					
GD 6–21	0.0 ± 0.0 (17)	0.4 ± 0.0 (12)	0.8 ± 0.0 (17)	1.6 ± 0.0 (12)	3.2 ± 0.1 (15)
LD 1–13	0.0 ± 0.0 (14)	0.8 ± 0.0 (12)	1.6 ± 0.0 (14)	3.4 ± 0.0 (12)	---

Statistical significance for an exposed group indicates a significant pairwise test compared to the vehicle control group. Statistical significance for the vehicle control group indicates a significant trend test.

*Statistically significant at p ≤ 0.05; * *p ≤ 0.01.

GD = gestation day; LD = lactation day.

^a^Data are presented as mean ± standard error (n). Statistical analysis performed by the Jonckheere (trend) and Shirley or Dunn (pairwise) tests. Water consumption values were excluded when excessive spillage was recorded.

^b^Note that lactation data represent water consumption by both dams and pups.

^c^Intake values not available due to early removal of this group.

^d^Statistical analysis was not performed on these data. Chemical intake values were excluded when excessive spillage was recorded.

Lactational water consumption (g/animal/day) by F_0_ dams was similar to that of control rats with the exception of the 12.5 mg/L group, where significantly increased water consumption (approximately 11–13% greater than the control group) was noted at all intervals during the lactation day (LD) 1–13 period (with the exception of the LD 1–4 interval). This resulted in a significant positive trend at all lactation intervals (Table 3). Chemical intake among exposure groups during lactation, estimated from water consumption, was generally proportional to exposure concentration; however, intake for the 12.5 mg/L group was slightly more than double that of the 6.25 mg/L group (Table 3). The estimated daily dose over the LD 1–13 interval was 0, 0.8, 1.6, and 3.4 mg thallium (I) sulfate/kg/day for the 0, 3.13, 6.25, and 12.5 mg/L groups, respectively.

#### 3.1.3 Maternal body weights

**Fig. 1:**
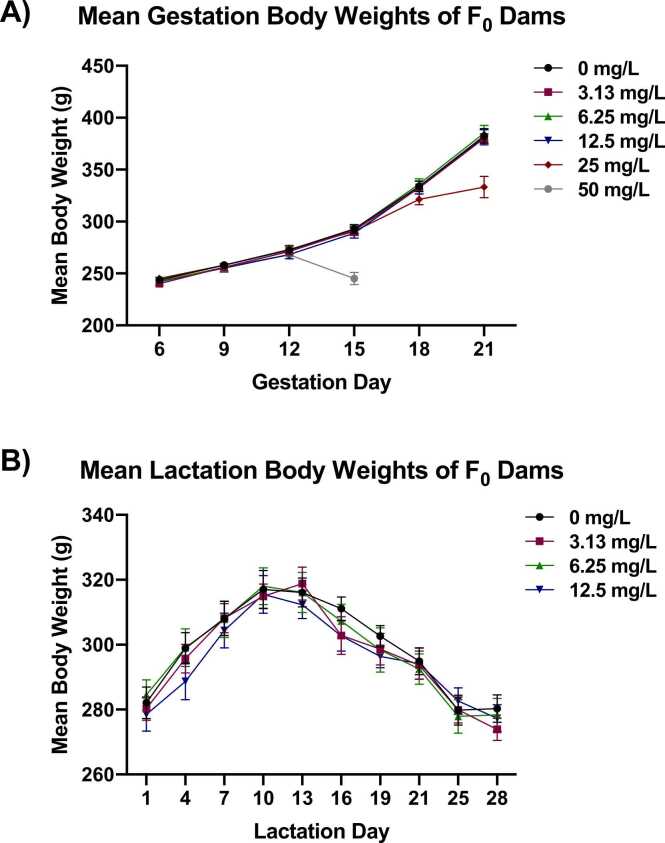
**Mean Body Weights of F**_**0**_**Dams during Gestation and Lactation.** Mean body weights of F_0_ dams during the perinatal period. Data are presented as mean ± SEM for gestation (A) and lactation (B). Information on statistical significance is available in CEBS.

Gestation body weights of dams exposed to 25 and 50 mg/L thallium (I) sulfate were significantly decreased relative to the control group when removed from the study (Fig. 1A). Body weights of dams in the 50 mg/L group were similar to those of the control group up to GD 12; between GD 12–15, dams in the 50 mg/L group displayed an approximate 9% decrease in absolute body weight (equating to an approximate 16% lower body weight relative to the control group on GD 15). This decrease in body weight, combined with the aforementioned clinical observations of overt toxicity, prompted removal of the 50 mg/L group from the study.

Body weights of dams in the 25 mg/L group were similar to those of the control group up to GD 18; by GD 21, body weights of the 25 mg/L group were significantly decreased (approximately 13% lower) relative to the control group. The decreased body weights in the 25 mg/L group were attributed to decreased body weight gains relative to the control group over the GD 18–21 interval (approximately 91% lower) and over the entire gestation period (GD 6–21; approximately 36%). Similar to the 50 mg/L group, the decrease in body weight combined with the early deaths and moribund removals prompted removal of the remaining dams in the 25 mg/L group from the study.

Body weights of all remaining exposure groups (3.13, 6.25, and 12.5 mg/L) were similar to those of the control group at all assessed gestational and lactational intervals (Fig. 1A and B).

#### 3.1.4 Pregnancy and littering

All dams on study were pregnant, including those in the 25 and 50 mg/L groups which were removed early. Exposure to thallium (I) sulfate did not affect reproductive performance parameters, including the percentage of pregnant females that produced pups, gestation length, live pups per litter, or pup sex distribution (data not shown).

#### 3.1.5 Pup findings

Clinical observations in pups were primarily limited to the 12.5 mg/L group (Table 4). Uneven hair growth at multiple sites (>3) was noted in all pups from all litters exposed to 12.5 mg/L during lactation (between postnatal day [PND] 11–24) which progressed to whole-body alopecia in all pups from all litters in the same exposure group between PND 18–28.

**Table 4:** Clinical Observations in Rat Pups Following Perinatal Exposure to Thallium (I) Sulfate^a^.

	0 mg/L	3.13 mg/L	6.25 mg/L	12.5 mg/L
Alopecia	0	0	0	159 (100.0)/12 (100.0) PND 18–28
Uneven Hair Growth, Multiple Sites (>3)	0	0	0	159 (100.0)/12 (100.0) PND 11–24

PND = postnatal day.

a Data are presented as the number of animals observed with clinical finding (%)/the number of litters from which those animals came from (%). Below the incidence are the days the clinical finding was observed.

**Fig. 2:**
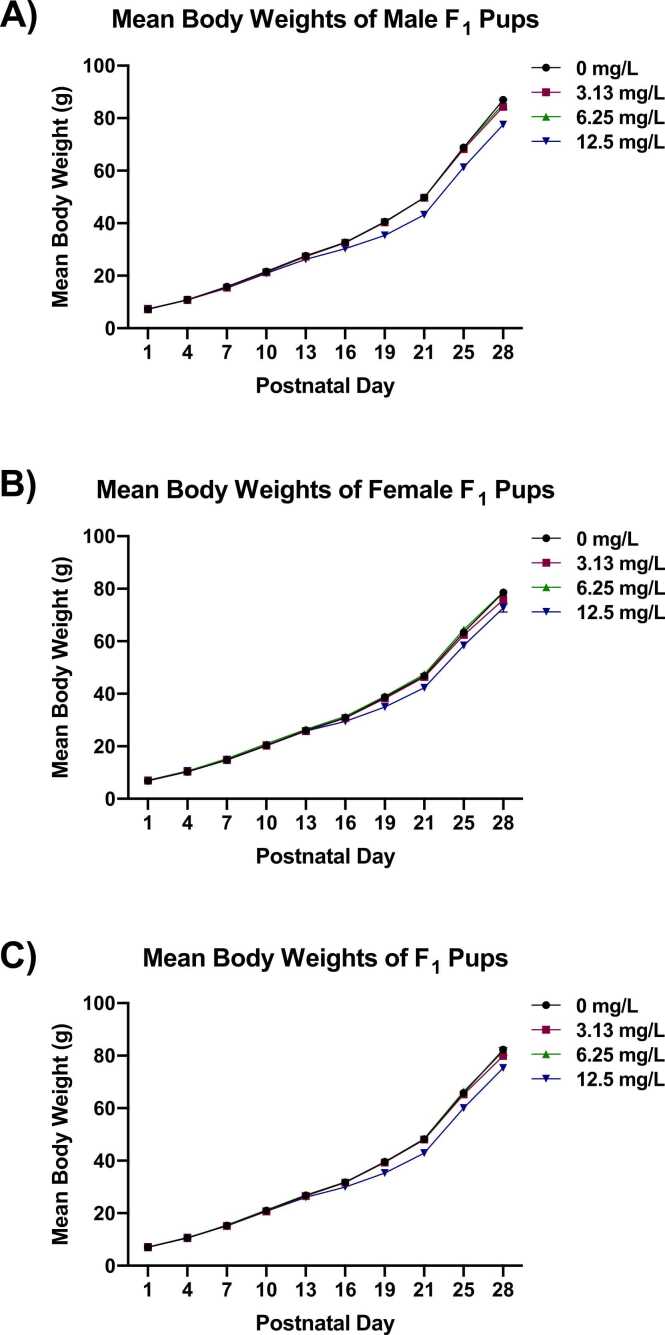
Mean Body Weights of F_1_ Pups during Gestation and Lactation. Mean body weights of F_1_ pups over the postnatal period. Data are presented as mean ± SEM for male pups (A), female pups (B), and all pups (C). Information on statistical significance is available in CEBS.

Body weight gains of all pups in the 12.5 mg/L group were significantly decreased relative to the control group during the PND 13–16, 16–19, and 19–21 intervals (approximately 11–32%) and were also significantly decreased over the full PND 4–28 interval (approximately 10%) (Table 5). Body weights of pups in the 12.5 mg/L group were significantly decreased (approximately 7–13%) relative to the control group starting on PND 16 (males) or PND 19 (females) (Fig. 2A and B). When data for all pups were combined, body weights of the 12.5 mg/L group were significantly decreased (approximately 6–11%) beginning on PND 16 (Fig. 2C).

**Table 5:** Mean Body Weight Gains of Rat Pups Following Perinatal Exposure to Thallium (I) Sulfate^a^.

PND	0 mg/L		3.13 mg/L		6.25 mg/L		12.5 mg/L	
	Weight (g)	N^b^	Weight (g)	N	Weight (g)	N	Weight (g)	N
Male and Female								
1–4	3.55 ± 0.10	221 (17)	3.44 ± 0.15	164 (12)	3.58 ± 0.09	223 (17)	3.58 ± 0.10	159 (12)
4–7	4.54 ± 0.16	180 (14)	4.58 ± 0.16	164 (12)	4.74 ± 0.19	186 (14)	4.40 ± 0.17	159 (12)
7–10	5.64 ± 0.18	179 (14)	5.60 ± 0.21	163 (12)	5.73 ± 0.14	184 (14)	5.53 ± 0.09	159 (12)
10–13	5.76 ± 0.10 *	178 (14)	5.76 ± 0.13	162 (12)	5.59 ± 0.13	182 (14)	5.39 ± 0.18	159 (12)
13–16	4.92 ± 0.08 * *	178 (14)	5.10 ± 0.14	162 (12)	5.00 ± 0.17	181 (14)^c^	3.85 ± 0.23 * *	159 (12)
16–19	7.90 ± 0.41 * *	178 (14)	7.67 ± 0.39	162 (12)	7.57 ± 0.44	181 (14)^c^	5.35 ± 0.25 * *	159 (12)
19–21	8.55 ± 0.12 * *	178 (14)	8.71 ± 0.18	162 (12)	8.87 ± 0.25	182 (14)	7.60 ± 0.31 *	159 (12)
21–25	17.67 ± 0.51	178 (14)	17.22 ± 0.31	162 (12)	18.02 ± 0.41	182 (14)	17.14 ± 0.63	159 (12)
25–28	16.48 ± 0.34	178 (14)	14.62 ± 0.49 *	162 (12)	15.27 ± 0.57	182 (14)	15.29 ± 0.51	159 (12)
4–28	71.56 ± 0.86 * *	178 (14)	69.30 ± 1.05	162 (12)	70.88 ± 1.43	182 (14)	64.55 ± 1.33 * *	159 (12)

Statistical significance for an exposed group indicates a significant pairwise test compared to the vehicle control group. Statistical significance for the vehicle control group indicates a significant trend test.

*Statistically significant at p ≤ 0.05; * *p ≤ 0.01. PND = postnatal day.

^a^Data are presented as mean ± standard error. Statistical analysis performed using linear mixed models with random litter effect for both trend and pairwise tests, using the Dunnett Hsu adjustment for multiple comparisons.

^b^Number of pups examined (number of litters examined).

^c^One female pup weight on PND 16 was excluded from analysis as an outlier.

#### 3.1.6 Internal concentration assessment

Internal thallium concentrations were evaluated in dams and their pups (Table 6). Compared to the control group, thallium concentrations increased with exposure concentration in all evaluated matrices at all time points; at GD 18, these increases were more than proportional to exposure concentration. GD 18 fetal and PND 4 pup thallium concentrations were similar to those of their corresponding dams, demonstrating considerable maternal transfer of thallium. There were no sex differences in thallium concentration in PND 4 pups.

**Table 6:** Summary of Thallium Concentrations in Rats Following Perinatal Exposure to Thallium (I) Sulfate in Drinking Water.

Thallium Concentration^a,b,c^	0 mg/L	3.13 mg/L	6.25 mg/L	12.5 mg/L	25 mg/L
**Gestation Day 18**					
Number Examined	3	---^d^	3	---	3
Dam Plasma (ng/mL)	0.202 ± 0.0791 * *	---	212 ± 14 *	---	1080 ± 62.4 * *
Dam RBCs (ng/mL)	BD^e^	---	19.3 ± 6.46	---	129 ± 19.0
Amniotic Fluid (ng/mL)	BD	---	56.0 ± 5.62	---	265 ± 10.4
Fetuses (ng/g)	4.32 ± 4.04 * *	---	390 ± 45.0 *	---	1860 ± 136 * *
**Postnatal Day 4**					
Number Examined	3	---	3	---	---
Dam Plasma (ng/mL)	0.162 ± 0.0337	---	214 ± 21.7 *	---	---
Dam RBCs (ng/mL)	BD	---	13.0 ± 1.69	---	---
Male Pup Plasma (ng/mL)	0.0958 ± 0.0265	---	218 ± 19.3 *	---	---
Female Pup Plasma (ng/mL)	0.0672 ± 0.0242	---	231 ± 24.1 *	---	---
Male Pup RBCs (ng/mL)	BD	---	9.90 ± 1.53	---	---
Female Pup RBCs (ng/mL)	BD	---	10.4 ± 2.13	---	---
**Postnatal Day 28**					
Number Examined	5	5	5	5	---
Dam Plasma (ng/mL)	0.196 ± 0.00567 * *	165 ± 11.1 * *	292 ± 29.0 * *	624 ± 26.5 * *	---
Dam RBCs (ng/mL)	BD	5.33 ± 0.683	8.13 ± 0.933	12.7 ± 1.17	---
Male Pup Plasma (ng/mL)	0.106 ± 0.0282 * *	93.6 ± 3.52 * *	191 ± 15.1 * *	430 ± 26.5 * *	---
Female Pup Plasma (ng/mL)	0.138 ± 0.0129 * *	97.5 ± 9.49 * *	198 ± 9.25 * *	394 ± 26.2 * *	---
Male Pup RBCs (ng/mL)	BD	4.84 ± 0.317	8.32 ± 1.25	12.7 ± 0.431	---
Female Pup RBCs (ng/mL)	BD	5.17 ± 0.249	8.76 ± 0.972	11.5 ± 1.02	---

Statistical significance for an exposed group indicates a significant pairwise test compared to the vehicle control group. Statistical significance for the vehicle control group indicates a significant trend test.

*Statistically significant at p ≤ 0.05; * *p ≤ 0.01. RBCs = red blood cells; BD = below detection.

^a^Dam and PND 28 pup data are presented as mean ± SEM (n), where n is the number of dams or pups. Statistical analysis for dam and PND 28 pup data performed by the Jonckheere (trend) and Shirley or Dunn (pairwise) tests.

^b^PND 4 pup data are presented as mean ± SEM of the litter means (n), where n is the number of litters. Statistical analysis for PND 4 pup data performed using a Datta-Satten modified Wilcoxon test.

^c^If over 20% of the animals in a group were above the limit of detection (LOD), then ½ the LOD value was substituted for values below the LOD.

^d^Data not recorded for this group and/or time point.

^e^When the control group did not have over 20% of its values above the limit of detection, no mean or standard error were calculated, and no statistical analysis was performed.

At study termination on PND 28, thallium concentrations in pup plasma were within twofold of concentrations in dam plasma and pup plasma concentrations were similar in both sexes. Thallium plasma concentrations in pups from the 6.25 mg/L group were similar at the PND 4 and PND 28 time points for both males (218 and 191 ng/mL, respectively) and females (231 and 198 ng/mL, respectively). Thallium appeared to preferentially partition into plasma relative to red blood cells (RBCs), as concentrations in plasma were 8- to 22-fold higher than those in RBCs in dams and pups at all time points.

Plasma concentrations in dams from the 6.25 mg/L group (the only exposed group with plasma concentrations measured at all three time points) were similar across the GD 18 (212 ng/mL), PND 4 (214 ng/mL), and PND 28 time points (292 ng/mL) (Table 6). However, consumed dose (estimated using mean chemical consumption) by the 6.25 mg/L dams throughout gestation (GD 6–21; approximately 0.8 mg/kg/day) was approximately half that consumed during lactation (LD 1–13; approximately 1.6 mg/kg/day), suggesting that dose-normalized plasma concentrations were in fact higher during gestation compared to lactation. Chemical consumption data, and subsequently the estimated consumed dose, for the LD 1–28 interval are not available, but the dose estimated for the LD 25–28 interval was approximately 4.4 mg/kg/day, indicating that relative plasma concentrations at the PND 28 time point were lower than those observed at both GD 18 and PND 4.

Low levels of thallium were detected in control plasma and fetus samples at all respective time points. The reason for this is unclear; however, it is not considered to have impacted the overall study findings.

### 3.2 2-week study in mice

#### 3.2.1 Viability and clinical observations

Mortality attributed to thallium (I) sulfate exposure occurred in male and female mice in the 100 mg/L group. Male and female mice in the 100 mg/L group were euthanized moribund (two males, two females) or found dead (three males, three females) between study day (SD) 6–8. All remaining mice in the 0, 6.25, 12.5, 25, and 50 mg/L groups survived to study termination at SD 14 (data not shown).

Clinical observations were limited to male mice in the 100 mg/L group and female mice in the 50 and 100 mg/L groups. Exposure-related observations were noted beginning on SD 6 (100 mg/L males), SD 8 (100 mg/L females), and SD 12 (50 mg/L females), and included labored or shallow breathing, hunched posture, lethargy, thinness, and red vaginal discharge. These observations generally coincided with indications of overt toxicity or were observed prior to mortality in the 100 mg/L groups (data not shown). Alopecia was not noted in any male or female mice.

#### 3.2.2 Water consumption and chemical intake

Water consumption (g/animal/day) by male mice over the SD 0–14 period was significantly increased in the 25 and 50 mg/L groups relative to control mice (Table 7). Significant increases over the shorter intervals were also noted for male mice in the 25 mg/L (SD 10–14) and 50 mg/L (SD 7–10, SD 10–14) groups; these increases were supported by the corresponding internal concentration data. There were no significant increases in water consumption by female mice over the SD 0–14 period, with sporadic increases and decreases noted at shorter intervals across most groups (Table 7). Chemical intake (mg thallium (I) sulfate/kg/day) was estimated from water consumption and was slightly more than proportional to exposure concentration for both males and females (Table 7). The estimated daily dose for male mice over the SD 0–14 interval was 0, 0.9, 2.1, 4.6, and 10.9 mg thallium (I) sulfate/kg/day for the 0, 6.25, 12.5, 25, and 50 mg/L groups, respectively. The estimated daily dose for female mice over the SD 0–14 interval was 0, 0.7, 1.7, 3.7, and 6.2 mg thallium (I) sulfate/kg/day for the 0, 6.25, 12.5, 25, and 50 mg/L groups, respectively.

**Table 7:** Water Consumption and Thallium (I) Sulfate Intake from Drinking Water during the Two-week Study in Mice.

	0 mg/L	6.25 mg/L	12.5 mg/L	25 mg/L	50 mg/L	100 mg/L
n	5	5	5	5	5	5
Water Consumption (g/animal/day)^a^						
**Males**						
SD 0–3	2.8 ± 0.5 *	3.1 ± 0.4	3.7 ± 0.2	3.1 ± 0.6	3.3 ± 0.2	4.0 ± 0.4
SD 3–7	3.2 ± 0.3	2.9 ± 0.5	3.7 ± 0.3	3.9 ± 0.3^b^	4.3 ± 0.4	3.6 ± 0.2^b^
SD 7–10	3.8 ± 0.4 * *	3.0 ± 0.7	4.1 ± 0.3	4.0 ± 0.9	5.4 ± 0.3 *	---^c^
SD 10–14	2.9 ± 0.5 * *	3.7 ± 0.1	3.7 ± 0.2	4.5 ± 0.7 *	4.7 ± 0.2 * *	---
SD 0–14	3.1 ± 0.3 * *	3.2 ± 0.1	3.8 ± 0.2	3.9 ± 0.2 *	4.4 ± 0.2 * *	---
**Females**						
SD 0–3	3.0 ± 0.0 *	1.7 ± 0.0 * *	2.6 ± 0.0	2.5 ± 0.0	2.2 ± 0.0 * *	2.3 ± 0.0 *
SD 3–7	2.1 ± 0.0	2.4 ± 0.0	2.3 ± 0.0	2.6 ± 0.0 * *	2.0 ± 0.0	2.5 ± 0.0 *
SD 7–10	2.9 ± 0.0 *	1.8 ± 0.0 * *	2.8 ± 0.0	2.5 ± 0.0	2.3 ± 0.0 * *	---
SD 10–14	2.5 ± 0.0	2.1 ± 0.0	3.0 ± 0.0	3.1 ± 0.0	2.0 ± 0.0	---
SD 0–14	2.6 ± 0.0	2.0 ± 0.0	2.7 ± 0.0	2.7 ± 0.0	2.1 ± 0.0	---
**Thallium (I) Sulfate Intake (mg/kg/day)**^**d**^						
**Males**						
SD 0–14	0.0 ± 0.0	0.9 ± 0.0	2.1 ± 0.1	4.6 ± 0.4	10.9 ± 0.6	---
**Females**						
SD 0–14	0.0 ± 0.0	0.7 ± 0.0	1.7 ± 0.0	3.7 ± 0.1	6.2 ± 0.2	---

Statistical significance for an exposed group indicates a significant pairwise test compared to the vehicle control group. Statistical significance for the vehicle control group indicates a significant trend test.

*Statistically significant at p ≤ 0.05; * *p ≤ 0.01. SD = study day.

^a^Data are presented as mean ± standard error. Statistical analysis performed by the Jonckheere (trend) and Shirley or Dunn (pairwise) tests. Water consumption values were excluded when excessive spillage was recorded.

^b^n = 4.

^c^Data not presented due to early removal of the animals from study.

^d^Statistical analysis was not performed on these data. Chemical intake values were excluded when excessive spillage was recorded.

#### 3.2.3 Body weights

**Fig. 3:**
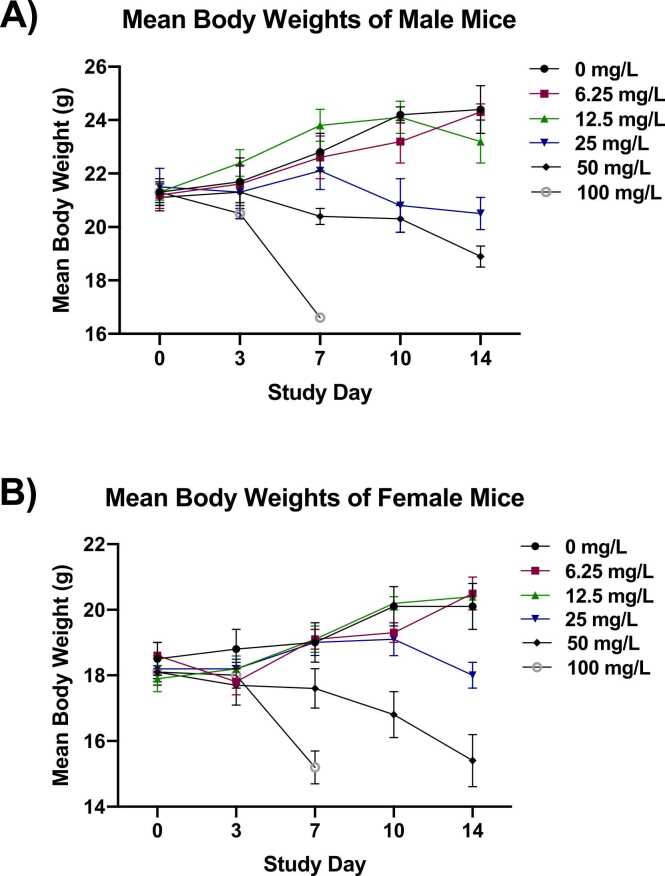
Mean Body Weights of Male and Female Mice during the Two-week Study. Mean body weights of mice over the 2-week study period. Data are presented as mean ± SEM for male mice (A) and female mice (B). Information on statistical significance is available in CEBS.

Male and female mice exposed to 25 mg/L thallium (I) sulfate displayed significantly decreased body weights, relative to the control group, beginning on SD 10 (males; approximately 14–16%) and SD 14 (females; approximately 11%) (Figs. 3A and 3B). Body weights of male and female mice in the 50 mg/L group were significantly decreased, relative to control mice, beginning on SD 7 (approximately 8–23%). Body weights of the 100 mg/L group at early termination (SD 7) were approximately 27% (males) and 20% (females) lower than those of the respective control group.

Male and female mice in the 25 and 50 mg/L groups lost weight over the 2-week study period (approximately 0.3–2.6 g; Table 8). In addition, body weight gain of male mice in the 12.5 mg/L group (1.9 g) was approximately 39% lower than that of the control group (3.1 g) over the study period. Between SD 0–7, prior to the early deaths, male and female mice in the 100 mg/L group also lost weight (4.1 g in males [n = 1] and approximately 3.0 g in females).

**Table 8:** Mean Body Weight Gains of Mice Exposed to Thallium (I) Sulfate for Two Weeks^a^.

	0 mg/L	6.25 mg/L	12.5 mg/L	25 mg/L	50 mg/L	100 mg/L
**n**	5	5	5	5	5	5
**Male (g)**						
SD 0–7	1.5 ± 0.6 * *	1.5 ± 0.9	2.4 ± 0.3	0.6 ± 0.3	-0.7 ± 0.2 * *	-4.1^b^
SD 7–14	1.6 ± 0.2 * *	1.7 ± 0.7	-0.6 ± 0.3 * *	-1.6 ± 0.2 * *	-1.5 ± 0.4 * *	---^c^
SD 0–14	3.1 ± 0.8 * *	3.2 ± 0.7	1.9 ± 0.5	-1.0 ± 0.3 * *	-2.2 ± 0.4 * *	---
**Female (g)**						
SD 0–7	0.5 ± 0.3 * *	0.5 ± 0.2	1.2 ± 0.2	0.8 ± 0.2	-0.5 ± 0.4	-3.0 ± 0.8 * *
SD 7–14	1.1 ± 0.2 * *	1.4 ± 0.4	1.3 ± 0.4	-1.1 ± 0.4 * *	-2.1 ± 0.3 * *	---
SD 0–14	1.6 ± 0.3 * *	1.9 ± 0.4	2.5 ± 0.4	-0.3 ± 0.5 * *	-2.6 ± 0.6 * *	---

Statistical significance for an exposed group indicates a significant pairwise test compared to the vehicle control group. Statistical significance for the vehicle control group indicates a significant trend test.

*Statistically significant at p ≤ 0.05; * *p ≤ 0.01. SD = study day.

^a^Data are presented as mean ± standard error. Statistical analysis performed by the Jonckheere (trend) and Williams or Dunnett (pairwise) tests.

^b^N = 1 due to early removal of animals from the 100 mg/L group.

^c^Data not presented due to early removal of the animals from study.

#### 3.2.4 Organ weights

Significant changes in organ weights were noted in male and female mice primarily in the 25 and 50 mg/L groups (Table 9). Absolute liver weights were significantly decreased in male mice in the 12.5, 25, and 50 mg/L groups (approximately 14%, 21%, and 40%, respectively) and in female mice in the 25 and 50 mg/L groups (approximately 17% and 33%, respectively). Relative liver weights were decreased in both males (approximately 22%) and females (approximately 13%) in the 50 mg/L group relative to the control group. Absolute and relative thymus weights were significantly decreased in both male and female mice in the 25 and 50 mg/L groups relative to the control group (approximately 37–57% lower in males and 24–50% lower in females). Increases in relative kidney weights (male and female mice) and relative testis weights (male mice) were also noted; however, these findings were not considered to be exposure related. Due to the significant decreases in terminal body weight in both males and females in the 25 and 50 mg/L groups, it is likely that many of the changes in relative organ weights in those groups were a result of the body weight effects and not directly related to exposure.

**Table 9:** Mean Organ Weights and Organ Weight-to-Body Weight Ratios of Mice Exposed to Thallium (I) Sulfate for Two Weeks^a,b^.

	0 mg/L	6.25 mg/L	12.5 mg/L	25 mg/L	50 mg/L
**n**	5	5	5	5	5
**Male**					
Necropsy Body Wt. (g)	24.4 ± 0.9 * *	24.3 ± 0.3	23.2 ± 0.8	20.5 ± 0.6 * *	18.9 ± 0.4 * *
Right Kidney					
Absolute (g)	0.22 ± 0.01 *	0.21 ± 0.01	0.21 ± 0.01	0.19 ± 0.01	0.20 ± 0.01
Relative (mg/g)^c^	9.19 ± 0.36 * *	8.79 ± 0.25	9.23 ± 0.28	9.47 ± 0.09	10.58 ± 0.16 * *
Left Kidney					
Absolute (g)	0.21 ± 0.01 *	0.20 ± 0.01	0.19 ± 0.01	0.18 ± 0.00 *	0.19 ± 0.01
Relative (mg/g)	8.76 ± 0.19 *	8.13 ± 0.31	8.37 ± 0.24	8.70 ± 0.13	9.93 ± 0.22 * *
Liver					
Absolute (g)	1.40 ± 0.10 * *	1.39 ± 0.05	1.21 ± 0.05 *	1.11 ± 0.04 * *	0.84 ± 0.04 * *
Relative (mg/g)	57.05 ± 2.17 * *	57.07 ± 1.43	52.32 ± 0.61	54.14 ± 0.31	44.61 ± 1.29 * *
Thymus					
Absolute (g)	0.069 ± 0.014 * *	0.065 ± 0.006	0.058 ± 0.007	0.030 ± 0.003 * *	0.033 ± 0.006 * *
Relative (mg/g)	2.81 ± 0.50 * *	2.68 ± 0.21	2.51 ± 0.29	1.46 ± 0.18 *	1.76 ± 0.33 *
Right Testis					
Absolute (g)	0.099 ± 0.002	0.096 ± 0.002	0.098 ± 0.002	0.097 ± 0.005	0.098 ± 0.002
Relative (mg/g)	4.09 ± 0.14 * *	3.96 ± 0.10	4.26 ± 0.16	4.73 ± 0.13 * *	5.20 ± 0.19 * *
Left Testis					
Absolute (g)	0.101 ± 0.003	0.093 ± 0.001	0.093 ± 0.002	0.093 ± 0.005	0.095 ± 0.002
Relative (mg/g)	4.15 ± 0.14 * *	3.84 ± 0.05	4.01 ± 0.15	4.52 ± 0.10	5.03 ± 0.20 * *
**Female**					
Necropsy Body Wt. (g)	20.1 ± 0.7 * *	20.5 ± 0.5	20.4 ± 0.1	18.0 ± 0.4 *	15.4 ± 0.8 * *
Right Kidney					
Absolute (g)	0.14 ± 0.01	0.15 ± 0.01	0.16 ± 0.01	0.15 ± 0.01	0.16 ± 0.01
Relative (mg/g)	7.04 ± 0.35 * *	7.51 ± 0.25	7.96 ± 0.28	8.58 ± 0.26 * *	10.49 ± 0.42 * *
Left Kidney					
Absolute (g)	0.13 ± 0.01	0.15 ± 0.01	0.14 ± 0.01	0.14 ± 0.00	0.15 ± 0.01
Relative (mg/g)	6.45 ± 0.36 * *	7.12 ± 0.25	7.07 ± 0.23	7.69 ± 0.24 *	9.66 ± 0.63 * *
Liver					
Absolute (g)	1.03 ± 0.08 * *	1.08 ± 0.04	1.05 ± 0.04	0.86 ± 0.04 *	0.69 ± 0.05 * *
Relative (mg/g)	51.17 ± 2.10 * *	52.41 ± 1.13	51.74 ± 1.94	47.95 ± 2.12	44.42 ± 1.50 *
Thymus					
Absolute (g)	0.084 ± 0.007 * *	0.078 ± 0.003	0.078 ± 0.004	0.057 ± 0.004 * *	0.042 ± 0.007 * *
Relative (mg/g)	4.14 ± 0.22 * *	3.81 ± 0.13	3.85 ± 0.17	3.16 ± 0.24 * *	2.68 ± 0.35 * *

Statistical significance for an exposed group indicates a significant pairwise test compared to the vehicle control group. Statistical significance for the vehicle control group indicates a significant trend test.

*Statistically significant at p ≤ 0.05; * *p ≤ 0.01.

^a^Data are presented as mean ± standard error. Statistical analysis performed by the Jonckheere (trend) and Williams or Dunnett (pairwise) tests.

^b^Data not presented for the 100 mg/L group due to early removal of the animals from study.

^c^Relative organ weights are given as mg organ weight/g body weight.

#### 3.2.5 Internal concentration assessment

Thallium concentrations were evaluated in mouse plasma and RBCs at the end of the 2-week exposure period (Table 10). Concentrations in mouse plasma increased with exposure concentration, and these increases were generally proportional to the corresponding increase in exposure concentration. The only exception was the 12.5 mg/L group, where concentrations increased approximately threefold compared to the 6.25 mg/L group. Thallium concentrations in plasma were consistently higher in males relative to females (approximately 37–58%). Thallium concentrations in RBCs generally increased with exposure concentration, particularly in the 25 and 50 mg/L groups, and tended to be higher in males relative to females (approximately 52% and 147% in the two highest exposure groups). The differences in male and female plasma and RBC concentrations were consistent with the corresponding chemical consumption data, where female mice consumed less than male mice across all exposure groups (Table 7). Similar to what was seen in rats, thallium appeared to preferentially partition into plasma relative to RBCs, with plasma concentrations approximately 11- to 33-fold higher than those in RBCs in both males and females. This further supports the idea that plasma may be a better matrix for biomonitoring studies.

**Table 10:** Summary of Thallium Concentrations in Mice Exposed to Thallium (I) Sulfate in Drinking Water for Two Weeks^a^.

Thallium Concentration^a,b^	0 mg/L	6.25 mg/L	12.5 mg/L	25 mg/L	50 mg/L
**Male**					
Number Examined^c^	5	4	5	5	5
Plasma (ng/mL)	0.245 ± 0.0941 * *	209 ± 11.8 *	627 ± 33.4 * *	1550 ± 48.5 * *	2730 ± 102 * *
RBCs (ng/mL)	BD^d^	14.4 ± 3.67	19.0 ± 7.53	55.4 ± 15.8	161 ± 45.0
**Female**					
Number Examined	5	5	4	5	4
Plasma (ng/mL)	0.0774 ± 0.0145 * *	132 ± 9.81 *^e^	443 ± 58.2 * *	1050 ± 58.6 * *	2000 ± 207 * *
RBCs (ng/mL)	BD	6.89 ± 2.51	41.7 ± 8.07	36.4 ± 11.0	65.3 ± 34.3

Statistical significance for an exposed group indicates a significant pairwise test compared to the vehicle control group. Statistical significance for the vehicle control group indicates a significant trend test.

*Statistically significant at p ≤ 0.05; * *p ≤ 0.01. RBCs = red blood cells; BD = below detection.

^a^Data are presented as mean ± SEM (n). Statistical analysis for dam data performed by the Jonckheere (trend) and Shirley or Dunn (pairwise) tests.

^b^If over 20% of the animals in a group were above the limit of detection (LOD), then ½ the LOD value was substituted for values below the LOD.

^c^Number of animals examined.

^d^When the control group did not have over 20% of its values above the limit of detection, no mean or standard error were calculated, and no statistical analysis was performed.

^e^n = 4.

Low levels of thallium were detected in control plasma from both male and female mice. The reason for this is unclear; however, it is not considered to have impacted the overall study findings.

## 4 Discussion

The studies reported here investigated the short-term toxicity of thallium (I) sulfate, a monovalent thallium salt, administered in drinking water to time-mated Sprague Dawley (Hsd:Sprague Dawley® SD®) rats and adult male and female B6C3F1/N mice. Drinking water was chosen as the route of exposure to mimic potential human exposure via contaminated groundwater, and time-mated rats were included to evaluate potential hazards following exposure during pregnancy and development. Adult mice were utilized as a representative model for exposure later in life.

Thallium is a heavy metal that is known to induce a broad spectrum of adverse health effects in humans including alopecia, neurotoxicity, and mortality following high dose acute poisoning events. In the studies reported here, rat dams and their offspring in the 25 and 50 mg/L exposure groups, along with mice exposed to 100 mg/L thallium (I) sulfate, were removed early due to moribundity, clinical signs of overt toxicity, and decreases in body weight relative to control animals. Regional alopecia was noted in 25 mg/L dams during gestation (beginning on gestation day [GD] 6) and in 12.5 mg/L dams during lactation (beginning on lactation day [LD] 6). Interestingly, alopecia was not noted in 50 mg/L dams during gestation, despite the early onset of the finding in 25 mg/L dams. The reason for this is unclear but may have been due to the underlying toxicity in the 50 mg/L dams. Additionally, uneven hair growth at multiple sites (>3) was noted in all pups from all litters exposed to 12.5 mg/L thallium (I) sulfate during the lactation period (between PND 11–24) which progressed to whole-body alopecia in all pups from all litters in the same exposure group between postnatal day (PND) 18–28. Interestingly, alopecia was not noted in mice, suggesting hair loss may be species-specific or correspond to increased sensitivity or potentially higher exposure concentrations at specific lifestages.

A 90-day study in adult Sprague Dawley rats administered thallium (I) sulfate by gavage reported statistically significant increases in the incidence of alopecia in male (0.04 mg/kg/day) and female (0.04 and 2 mg/kg/day) rats [10]. A number of the incidences reported in female rats were attributed to barbering behavior and the normal cycle of hair growth, making the relationship to thallium (I) sulfate exposure unclear. In the studies reported here, alopecia was noted in dams exposed to 12.5 and 25 mg/L thallium (I) sulfate, where chemical intake during lactation was estimated at 3.4 mg/kg/day for the 12.5 mg/L group and chemical intake during gestation was estimated at 3.2 mg/kg/day for the 25 mg/L group. These estimated daily doses are higher than those where alopecia was noted in the [10] report, but this may have been due to the differences in exposure length and use of adult rats (EPA) instead of pregnant rats (current study).

The effects of maternal thallium exposure on developing offspring have not been well-studied, but thallium has been shown to cross the placental barrier in mammals, including humans [42], [43], [8]. Measurement of thallium concentrations in dam plasma, amniotic fluid, fetuses (GD 18), and pup plasma (PND 4) in the studies reported here indicated maternal transfer of thallium to offspring during gestation and lactation. A literature review by Hoffman (2000) [23] summarized case reports of thallium poisoning during pregnancy, including five women each exposed during the first or second trimester and eight women exposed during the third trimester. Alopecia was reported in one child each exposed during the first or second trimester and four children exposed during the third trimester. Thallium concentrations in these children were not consistently measured across the different cases, so the incidences of alopecia cannot be tied to a specific in utero exposure level. Data from the 2015–2016 National Health and Nutrition Examination Survey reported mean urinary thallium concentrations (corrected for creatinine) in the general population (n = 3058) of 0.171 µg/L (95% CI: 0.163–0.181 µg/L). Mean urinary thallium concentrations in children 3–5 years old (n = 485) were 0.344 µg/L (95% CI: 0.330–0.358 µg/L) [7]. Although urinary concentrations of thallium were not measured in the current study, urinary measurements were included in the subsequent 90-day studies to generate data that allow for direct comparison to human urinary levels.

When looking at thallium concentrations across the different matrices evaluated, concentrations in plasma tended to be higher than those in red blood cells (RBCs), suggesting that thallium preferentially partitions into plasma rather than RBCs. This further supports the idea that plasma may be a better matrix for biomonitoring studies and was factored into the design of the follow-on subchronic studies. Interestingly, low levels of thallium were observed in some of the control samples, including plasma (rat dams and pups at all time points; male and female mice) and fetuses (at GD 18). Since the concentrations noted in control samples were sporadic, it is likely that these estimated values arose from the analytical assay.

When comparing between rats and mice exposed to 6.25 mg/L thallium (I) sulfate, and normalizing for consumed dose, relative mean plasma thallium concentrations were similar between male mice and GD 18 dams, and female mice and PND 4 dams, respectively; concentrations in male mice and GD 18 dams were higher than those in female mice and PND 4 dams. Male mice consumed a daily dose similar to that of dams during gestation (approximately 0.9 and 0.8 mg/kg/day, respectively); however, the daily dose consumed by female mice was approximately half that consumed by dams during lactation (approximately 0.7 and 1.6 mg/kg/day, respectively). Looking across sexes, plasma concentrations in male mice were consistently higher than those in female mice, and this was consistent with the corresponding chemical consumption data, where female mice consumed a lower dose than male mice at all exposure concentrations. This trend remained consistent when plasma concentrations were normalized to consumed dose, with the exception of the 50 mg/L groups, where relative plasma concentrations were actually higher in female mice than in males. These data suggest that there may be both species- and sex-specific differences in the absorption, distribution, metabolism, and excretion of thallium.

Exposure to thallium (I) sulfate at concentrations ≤ 12.5 mg/L did not impact F_0_ dam body weights, maintenance of pregnancy, littering parameters, or F_1_ survival (PND 4–28). Mean body weights of pups in the 12.5 mg/L group were significantly decreased relative to the control group starting on PND 16 (males) or PND 19 (females). Mean body weight gains of all pups were significantly decreased during the PND 13–16, PND 16–19, and PND 19–21 intervals and were also significantly decreased over the full PND 4–28 interval. While the decreases at individual postnatal intervals were fairly significant, the decrease over the entire postnatal interval remained within 10% of control animals and did not impact pup survival. In mice, significant decreases in body weight were noted in males and females in the 25 and 50 mg/L groups. Both male and female mice in the 25 and 50 mg/L groups also lost weight over the 2-week study period. The lack of body weight effects in surviving F_0_ dams is intriguing, particularly when effects were noted in mice as early as study day (SD) 7 (50 mg/L) and SD 10 (25 mg/L). This was likely due to differences in consumed dose, as mice generally consumed a higher daily dose (mg/kg/day), than did rat dams at the same exposure concentrations, due to higher proportional water consumption. These data further support the potential for lifestage- and/or species-specific effects of thallium (I) sulfate exposure.

Significant decreases in liver weight were noted in both male and female mice primarily in the 25 and 50 mg/L groups. Effects of thallium (I) sulfate on liver weight have not been consistently reported; however, multiple studies report other liver effects including changes in triglyceride levels, lipid peroxidation, glutathione levels, and serum alkaline phosphatase, alanine aminotransferase, aspartate aminotransferase, and bilirubin levels [46], [44], [45]. Increases in relative kidney weight were also noted; however, this finding was likely a result of changes in body weight and not directly related to exposure. While findings in the kidney were not considered exposure-related in the studies reported here, thallium has been shown to readily accumulate in the kidney following intraperitoneal injection [47], [48], and increases in kidney weight were previously noted in studies of Wistar albino rats exposed to thallium (I) acetate or thallium (III) oxide in the diet for 15 weeks [49]. Leloux et al. (1987) [50] also reported increases in absolute kidney weight in male and female rats following a single gavage dose (20 mg/kg) or four daily gavage doses (1 mg/kg/day) thallium (I) nitrate. Relative testis weights were also significantly increased in the 25 mg/L and 50 mg/L groups, which was consistent with previously reported testicular effects following thallium (I) sulfate exposure [25], [26], [51], [27].; however, the toxicological relevance of this finding is unclear.

## 5 Conclusions

In conclusion, thallium (I) sulfate concentrations ≤ 12.5 mg/L in rats, and ≤ 25 mg/L in mice, were well tolerated. The main findings from these studies included whole body alopecia in F_1_ rat pups and significantly decreased body weights for both rats and mice. Data from these short-term toxicity studies were used to inform the design and conduct of subchronic (90-day) studies of thallium (I) sulfate, including neurobehavioral evaluations (https://ntp.niehs.nih.gov/go/ts-16019), and will help to provide additional data needed to evaluate the potential public health risk of thallium (I) sulfate exposure.

## CRediT authorship contribution statement

**Kelly A. Shipkowski:** Conceptualization, Methodology, Writing – original draft, Writing – review & editing. **Troy D. Hubbard:** Conceptualization, Methodology, Writing – original draft, Writing – review & editing. **Kristen Ryan:** Methodology**,** Writing – review & editing**. Suramya Waidyanatha:** Conceptualization, Methodology, Writing – review & editing. **Helen Cunny:** Methodology, Writing – review & editing. **Keith R. Shockley:** Methodology, Formal analysis, Writing – review & editing. **Joshua L. Allen:** Investigation, Writing – review & editing, Supervision. **Heather Toy:** Investigation, Writing – review & editing, Supervision. **Keith Levine:** Investigation, Writing – review & editing. **James Harrington:** Investigation, Writing – review & editing. **Laura Betz:** Formal analysis, Writing – review & editing. **Barney Sparrow:** Investigation, Writing – review & editing, Project administration. **Georgia K. Roberts:** Conceptualization, Methodology, Writing – review & editing, Project administration.

## Declaration of Competing Interest

The authors declare that they have no known competing financial interests or personal relationships that could have appeared to influence the work reported in this paper.

## Data Availability

All data are available in the Chemical Effects in Biological Systems (CEBS) database: https://cebs.niehs.nih.gov/cebs/.
