# Supplementary material for: Short-term toxicity studies of thallium (I) sulfate administered in drinking water to Sprague Dawley rats and B6C3F1/N mice
Source: Toxicol Rep. 2023 May 11;10:621–32. doi: 10.1016/j.toxrep.2023.05.003 (PMC10209484; doi:10.1016/j.toxrep.2023.05.003)
Supplement: Supplementary file 1 — Supplementary material [file mmc1.docx]

**SUPPLEMENTAL MATERIAL**

**Quantitation of Total Thallium in Biological Matrices**

Total thallium concentration was quantitated using an inductively coupled plasma-mass spectrometry (ICP-MS) method. Thallium stock solutions (10 µg/mL) were procured from High Purity Standards (Charleston, SC) and Inorganic Ventures (Christiansburg, VA). Praseodymium (1,000 µg/mL) to be used as an internal standard was obtained from High Purity Standards (Charleston, SC). HNO_3_ (70%, trace metal grade) was obtained from Fisher Scientific (Hampton, NH) and H_2_O_2_ (30%, trace metal grade) was obtained from Ricca Chemical Company (Arlington, TX). The following rat matrices were purchased from BioIVT (Westbury, NY): Sprague Dawley rat plasma; male Hsd:Sprague Dawley®SD® (HSD) rat red blood cells; HSD gestation day (GD) 18 maternal rat plasma, fetuses, and amniotic fluid; HSD postnatal day (PND) 7 maternal and pup plasma; B6C3F1/N mouse plasma.

All standards and samples were prepared in deionized water. A 1.00 µg/mL praseodymium stock solution was prepared by diluting 1,000 µg/mL in 1% (v/v) HNO_3_. Thallium solutions at target concentrations of 0.01, 0.10, and 1.0 µg/mL were prepared by diluting 10 µg/mL stock standards with HNO_3_ in polypropylene tubes in a clean, high-efficiency particulate air (HEPA)-filtered plastic fume hood to a final HNO_3_ concentration of 20%. Two sets of these spiking solutions were prepared using the two commercially procured stock standards of thallium.

Matrix calibration standards and quality control (QC) standards were prepared in Sprague Dawley rat plasma by transferring 100 µL aliquots to 15-mL polypropylene tubes in a clean hood. Thallium spiking standards were added to produce matrix calibration standards at target concentrations of 1.25, 2.5, 5, 50, 100, 250, and 500 ng/mL plasma. Matrix QC samples (n = 9/concentration) were prepared similarly at 5 and 40 ng/mL in Sprague Dawley rat plasma and all representative study matrices.

To all standards 1.00 mL of 70% HNO_3_ was added and heated uncapped at 95°C for 30 minutes in SCP (Quebec, Montreal, Canada) DigiPrep graphite heating block. After cooling to room temperature, a 0.500 mL aliquot of 30% H_2_O_2_ solution was added to each tube and samples were returned to the block for an additional 30 minutes at 95 °C. Samples were allowed to cool to room temperature, 0.0250 mL of a 1.00 μg/mL solution of praseodymium was added and diluted to a final volume of 5.00 mL with deionized water prior to analysis by ICP-MS.

Study plasma, amniotic fluid, and red blood cell samples were prepared similarly for analysis without the addition of thallium spiking standards. Five individual fetuses were selected from each litter, thawed at room temperature, and homogenized with a Brinkmann Polytron prior to preparing the same way as for other study matrices for determination of thallium concentration.

All standards and samples were analyzed for total thallium concentration using an X-Series II quadrupole ICP-MS (Thermo Fisher Scientific, Bridgewater, NJ) equipped with a Peltier-cooled spray chamber and an ASX-500 autosampler (Teledyne CETAC Technologies, Omaha, NE). Thallium isotope Tl^205^ and praseodymium (Pr^141^) was monitored for quantitation. The ratio of Tl^205^ signal in each standard or sample to the Pr^141^ signal versus the thallium concentration was related using a linear least-squares regression. Thallium concentration in samples were calculated using the calibration equation and the dilution factors arising from the sample digestion process.

Plasma calibration curves were linear with a correlation coefficient r>0.99. The accuracy estimated as percent relative error (%RE) was ≤±8.0% at all standard levels. The lower limit of quantitation (LOQ) was 1.25 ng/mL. The limit of detection (LOD) was calculated as three times the standard deviation of the determined concentration for six replicates of the LOQ. The estimated LOD was 0.086 ng/mL.

The precision (estimated as percent relative standard deviation, %RSD) and accuracy of the method were determined using Sprague Dawley rat plasma QC samples prepared at two concentrations. The estimated %RE and %RSD were ≤±13.0% and ≤0.7%. Estimated %RE and %RSD for all study matrices (GD 18 maternal plasma, amniotic fluid, and fetus; PND 7 maternal and pup plasma; B6C3F1/N mouse plasma; rat blood cells) were <±5.2% and ≤5.9%, respectively, demonstrating the suitability of the assay to quantitate the concentration of thallium in study samples. QC samples run with each batch of study samples were ≤±10% of the nominal, demonstrating the accuracy of the study sample concentrations.
